# Structural Properties of Zinc Oxide Nanorods Grown on Al-Doped Zinc Oxide Seed Layer and Their Applications in Dye-Sensitized Solar Cells

**DOI:** 10.3390/ma7042522

**Published:** 2014-03-28

**Authors:** Kyung Ho Kim, Kazuomi Utashiro, Yoshio Abe, Midori Kawamura

**Affiliations:** Department of Materials Science and Engineering, Kitami Institute of Technology, 165 Koen-cho, Kitami, Hokkaido 090-8507, Japan; E-Mails: m1252600026@std.kitami-it.ac.jp (K.U.); abeys@mail.kitami-it.ac.jp (Y.A.); kawamumd@mail.kitami-it.ac.jp (M.K.)

**Keywords:** ZnO, nanorods, Al-dopant, annealing temperature, dye-sensitized solar cells

## Abstract

We fabricated zinc oxide (ZnO) nanorods (NRs) with Al-doped ZnO (AZO) seed layers and dye-sensitized solar cells (DSSCs) employed the ZnO NRs between a TiO_2_ photoelectrode and a fluorine-doped SnO_2_ (FTO) electrode. The growth rate of the NRs was strongly dependent on the seed layer conditions, *i.e*., thickness, Al dopant and annealing temperature. Attaining a large particle size with a high crystallinity of the seed layer was vital to the well-aligned growth of the NRs. However, the growth was less related to the substrate material (glass and FTO coated glass). With optimized ZnO NRs, the DSSCs exhibited remarkably enhanced photovoltaic performance, because of the increase of dye absorption and fast carrier transfer, which, in turn, led to improved efficiency. The cell with the ZnO NRs grown on an AZO seed layer annealed at 350 °C showed a short-circuit current density (*J*_SC_) of 12.56 mA/cm^2^, an open-circuit voltage (*V*_OC_) of 0.70 V, a fill factor (*FF*) of 0.59 and a power conversion efficiency (PCE, η) of 5.20% under air mass 1.5 global (AM 1.5G) illumination of 100 mW/cm^2^.

## 1. Introduction

Zinc oxide (ZnO) is one of the most attractive II–VI semiconductor oxide materials, because of its wide resistivity range (10^−4^–10^12^ Ω·cm), direct wide band gap (3.37 eV) and large exciton binding energy (60 meV) at room temperature [1–5]. The existence of various ZnO nanostructures (nanoparticles, nanowires, nanorods, plate-like, flower-like, *etc*.) has expanded their use in many devices, such as thin film transistors (TFTs), field emission devices, solar cells, chemical sensors and acoustic devices [3–6].

In general, the role of dopants (Al, Co, Li, *etc*.) on the growth of the ZnO nanorods (NRs) during hydrothermal synthesis has been well reported [7–9]. Fang *et al.* reported that the length of Al-doped ZnO NRs increased, whereas their diameter decreased with the incorporation of Al [7]. Caglar *et al.* reported that the sheet resistivity of Co-doped ZnO NRs decreased compared to that of undoped ZnO NRs. However, the crystallinity of the NRs deteriorated with the addition of Co [8]. Kung *et al.* reported that the length of Li-doped ZnO NRs increased with an increase in the thickness of the sputtered undoped ZnO seed layer [9].

It was also reported that the crystallinity, surface morphology, thickness and preparation conditions of the undoped ZnO seed layer played an important role on the growth of the ZnO NRs [10–14]. Dopants were well known as an effective method of modifying the physical characteristics of the ZnO seed layers [15,16]. However, the effects of the doped ZnO seed layer on the structural properties of the ZnO NRs have rarely been reported. In this study, we investigated the structural properties of ZnO nanostructures (nanorods, plate-like, flower-like) grown on an Al-doped ZnO (AZO) seed layers prepared by a sol-gel solution process on different substrate materials (glass and fluorine-doped SnO_2_ (FTO) coated glass). The sol-gel method has the advantage of being a low-cost and simple method of preparing a large-area thin film with excellent compositional control [17–19]. Compared to ZnO NRs grown on an undoped ZnO seed layer, the length and alignment of ZnO NRs grown on the AZO seed layer significantly improved. The properties of ZnO NRs could be modified by the incorporation of an Al dopant on the ZnO seed layers.

Furthermore, there were several research studies showing great interest in synthesizing ZnO NRs, which were composed of TiO_2_ nanoparticles (NPs) or ZnO NPs, in the dye-sensitized solar cells (DSSCs) [20–23]. ZnO NRs with a large surface area and fast electron transport rate could provide the improved photovoltaic performance of DSSCs. In this paper, the photovoltaic characteristic of DSSCs employing the ZnO NRs located between the TiO_2_ and FTO electrodes is also discussed. The DSSC with ZnO NRs grown on a AZO seed layer annealed at 350 °C showed better performance (5.20%) in comparison with the cell with only TiO_2_ (3.49%). Thus, the introduction of ZnO NRs grown on an AZO seed layer as the photoelectrode provides an alternative method of improving the photovoltaic performance of DSSCs.

## 2. Results and Discussion

**Figure 1.**
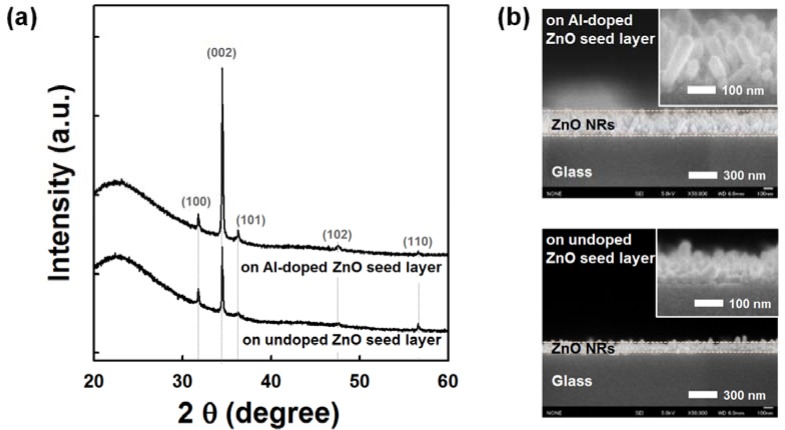
(**a**) XRD patterns and (**b**) cross-sectional field emission scanning electron microscopy (FESEM) images of ZnO nanorods (NRs) grown on undoped and Al-doped ZnO seed layer (20 nm-thick).

Figure 1a shows the XRD patterns of the ZnO NRs grown on undoped and AZO seed layers annealed at 150 °C. All samples show the hexagonal wurtzite structure of ZnO (Joint Committee on Powder Diffraction Standards (JCPDS) card No. 36-1451). ZnO NRs grown on the AZO seed layer show a stronger diffraction peak corresponding to the (002) plane growing along the *c*-axis perpendicular to the substrate surface; weak (100), (101), (102) and (110) peaks are also observed. With the addition of the Al dopant on the ZnO seed layer, the increased (002) peak intensity and decreased full-width at half-maximum (FWHM) indicate an improvement in the crystallinity of the NRs grown on the AZO seed layer. These results are consistent with the following field emission scanning electron microscopy (FESEM) observations. As shown in Figure 1b, the average length of NRs grown on the AZO seed layer was approximately 200 nm, which is two times longer than NRs grown on the undoped ZnO seed layer. The average diameters of NRs grown on the Al-doped and undoped ZnO seed layers were 50 nm and 40 nm, respectively. It is clear that the growth rate of the ZnO NRs increases with the incorporation of the Al dopant on the ZnO seed layer. It is well known that catalyst metals (Au, Ag, Pt, *etc*.) on the ZnO seed layer improve the growth of ZnO NRs [24]. The Al dopant might act as a catalyst for enhancing the growth of ZnO NRs. The Zn/O ratio of AZO film is found to be close to one, as listed in Table S1 of the Supplementary Information. By using a sputter deposition method, it was reported that the diameter and density of the ZnO NRs were strongly influenced by the ZnO seed layer preparation condition (Zn/O atomic ratio) [25]. However, the influence of the chemical properties of the AZO seed layer on the growth of NRs still needs further study. The growth of well-aligned NRs at low temperature has significant potential application in nanoscale electronic and optoelectronic devices on flexible polymer substrates.

**Figure 2.**
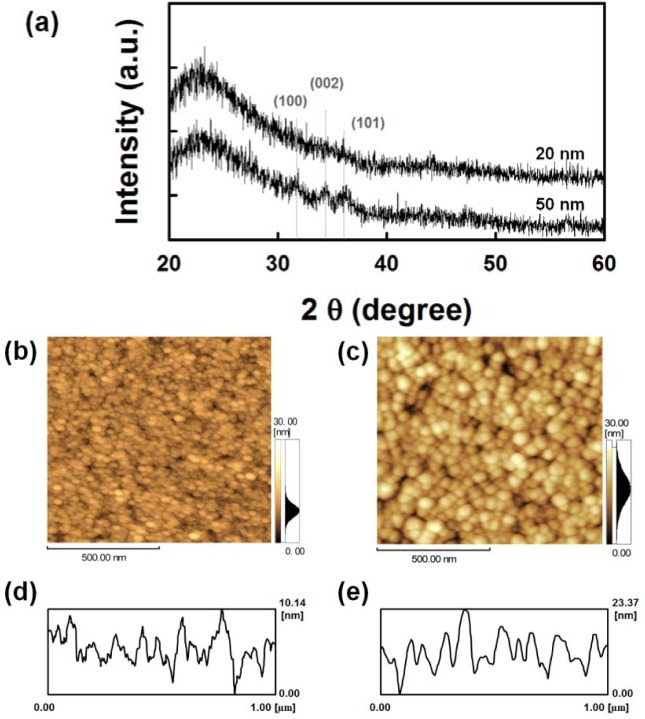
(**a**) XRD patterns and (**b**–**e**) atomic force microscopy (AFM) images and corresponding line roughness profiles of Al-doped ZnO (AZO) seed layers on glass substrates: (**b**,**d**) 20-nm-thick and (**c**,**e**) 50-nm-thick seed layer. The seed layer annealing temperature is 150 °C.

To further investigate the effect of the AZO seed layer on the growth of ZnO NRs, the structural and morphological properties of the seed layers with different thicknesses were studied. From the XRD patterns shown in Figure 2a, the 20 nm-thick AZO layer shows an amorphous phase, and diffraction peaks corresponding to the (100), (002) and (101) orientation appear with increasing thickness of the film (50 nm-thick). The peak intensity and crystalline size are associated with the film thickness [26,27]. Figure 2b–e show the two-dimensional (2D) atomic force microscopy (AFM) images and corresponding line profiles of the surfaces of the AZO seed layers with varying thicknesses. With an increase in the film thickness, the average particle size increases, resulting in an increase of the root mean square (RMS) roughness from 1.9 nm (20 nm-thick) to 4.7 nm (50 nm-thick), but the corresponding line roughness frequency decreases [12].

**Figure 3.**
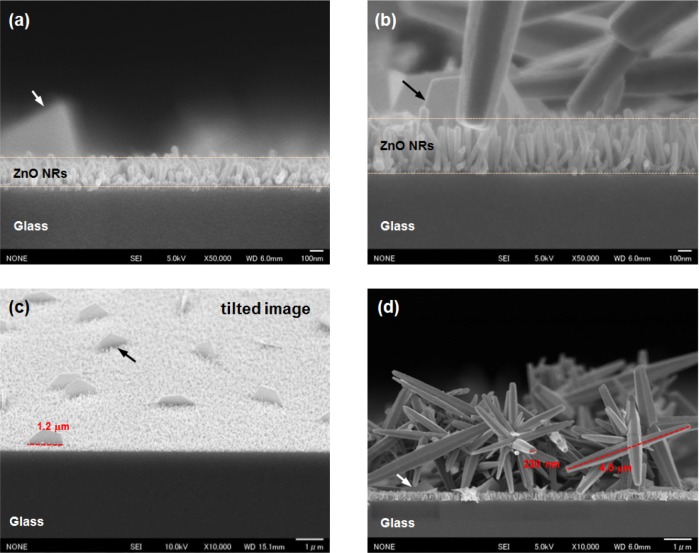
FESEM images of ZnO NRs grown on (**a**,**c**) 20 nm-thick and (**b**,**d**) 50 nm-thick AZO seed layers annealed at 150 °C; (**a**,**b**) under high magnification and (**c**,**d**) under low magnification. Arrows indicate plate-like ZnO structures.

Figure 3 shows the cross-sectional FESEM images of the ZnO NRs grown on 20 nm-thick (Figure 3a,c) and 50-nm-thick (Figure 3b,d) AZO seed layers annealed at 150 °C. The average length of the NRs clearly increased with the seed layer thickness, from 200 nm for the 20 nm-thick layer to 350 nm for the 50 nm-thick layer, as shown in Figure 3a,b, respectively. The figure also shows the good alignment of the ZnO NRs, with a perpendicular orientation to the substrate with increasing seed layer thickness. These results are consistent with those of other reports [9,14]. The alignment and length of the NRs could improve with the larger particle size of the ZnO seed layer (Figure 2b,c). The alignment and length are essential characteristics of the NRs, and these characteristics play a determining role in the properties of the NRs. Interestingly, plate-like ZnO structures are randomly distributed on the surfaces of the ZnO NRs, as shown in Figure 3c. The plates are a hexagonal (half-obscured) and are ~1.2 μm in diameter and ~80 nm in width. The plate-like ZnO structures are also observed on the surfaces of the ZnO NRs grown on the undoped ZnO seed layer. The ZnO NRs on the undoped and AZO seed layers have a plate density of 0.01 and 0.05 per μm^2^, respectively. The addition of an Al dopant on a ZnO seed layer also leads to an increase in plate density on the surface of the ZnO NRs. With the 50 nm-thick AZO seed layer, plate-like and the flower-like ZnO structures co-exist on the surfaces of the vertical ZnO NRs, as shown in Figure 3d. The diameter and width of the plate-like structures are less variable with increasing seed layer thicknesses. The length and diameter of the flower-like ZnO structures assembled around dozens of hexagonal-shaped rods are ~4.5 μm and ~230 nm, respectively. It was reported that the incorporation of Co or Al during the synthesis of the ZnO NR solution formed several distinct ZnO nanostructures [8,28].

**Figure 4.**
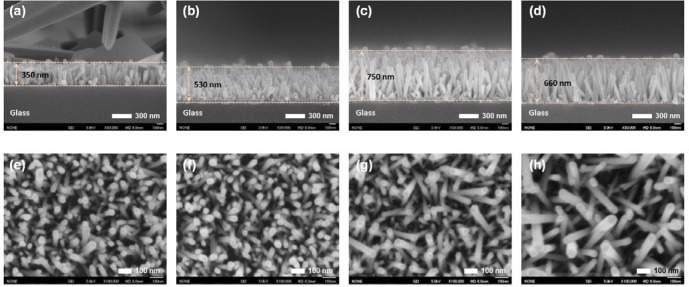
(**a**–**d**) Cross-sectional and (**e**–**h**) plan-view FESEM images of ZnO NRs grown on 50 nm-thick AZO seed layers; (**a**,**e**) 150 °C, (**b**,**f**) 250 °C, (**c**,**g**) 350 °C and (**d**,**h**) 450 °C.

**Figure 5.**
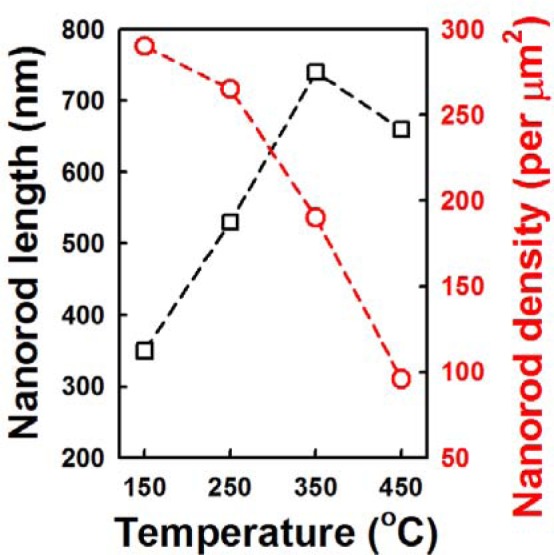
The average length (rectangular) and density (circle) of ZnO NRs with various seed layer annealing temperatures.

**Figure 6.**
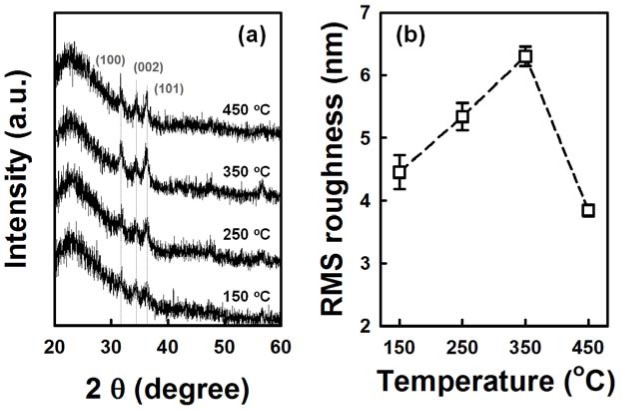
(**a**) XRD patterns and (**b**) root mean square (RMS) roughness of AZO seed layers with various seed layer annealing temperatures.

Figure 4 shows the cross-sectional and plan-view FESEM images of the ZnO NRs grown on AZO seed layers fabricated at various annealing temperatures from 150 °C to 450 °C. The variation of seed layer annealing treatment leads to a remarkable change in the growth rate of the ZnO NRs. With an increase in the annealing temperature to 350 °C, the length of the ZnO NRs increases to 750 nm, and then, it decreases with further increases of annealing temperature. The XRD patterns of the ZnO NRs grown on the AZO with seed annealing temperatures are shown in Figure S1. NRs grown on the AZO annealed at 350 °C shows higher relative intensity of the (002) peak. Length and density of the NRs are plotted in Figure 5. The ZnO NRs possess a hexagonal cross-section, as shown in the plan-view FESEM images. With increasing seed layer annealing temperature, the average diameter of the NRs gradually increases, and the density of the NRs decreases somewhat, as shown in Figure 5. It was reported that the growth rate of ZnO NRs on a sputtered ZnO seed layer increased with increasing annealing temperature, because of the improved crystallinity of the seed layer [14]. From the XRD patterns of the seed layers, shown in Figure 6a, the intensity of the diffraction peaks increases with increasing seed layer annealing temperature to 350 °C, and then, it slightly decreases. The surface roughness shows the same tendency in the XRD results as the annealing temperature (Figure 6b). These results well support the claim that the optimum annealing temperature of a seed layer with good crystallinity and surface morphology leads to well-aligned growth of the ZnO NRs.

Wahid *et al.* discussed the nanostructures of ZnO NRs with seed layer annealing temperatures ranging from 100 to 200 °C. The growth transition from vertically grown ZnO NRs to homocentric ZnO bundling occurred due to the agglomeration of nanoparticles in the seed layer during annealing (above 170 °C) [29]. In our case, the plate-like and flower-like structures on the surfaces of the vertical ZnO NRs disappeared with increasing seed layer annealing temperatures, which might be due to the decrease in the strain in the nanoparticles in the seed layer, owing to better crystallinity with increasing seed layer annealing temperature. However, further investigation is needed on the relation between the growth transition mechanism and the chemical properties of the AZO seed layer, because both the physical properties and the chemical components of AZO change significantly with annealing treatments [3]. The typical synthesis process of the ZnO NRs in an aqueous solution of zinc nitrate and hexamethylenetetramine (HMT) was based on the following reaction [30].

$$\text{Zn}{\left({\text{NO}}_{3}\right)}^{2}\to {\text{Zn}}^{2+}+2{\text{NO}}_{3}{}^{-}$$ (1)

$${\text{C}}_{6}{\text{H}}_{12}{\text{N}}_{4}+6{\text{H}}_{2}\text{O}↔6\text{HCHO}+4{\text{NH}}_{3}$$ (2)

$${\text{NH}}_{3}+{\text{H}}_{2}\text{O}↔{\text{NH}}_{4}{}^{+}+{\text{OH}}^{-}$$ (3)

$${\text{Zn}}^{2+}+2{\text{OH}}^{-}↔\text{Zn}{\left(\text{OH}\right)}_{2}$$ (4)

$$\text{Zn}{\left(\text{OH}\right)}_{2}↔\text{ZnO}+{\text{H}}_{2}\text{O}$$ (5)

**Figure 7.**
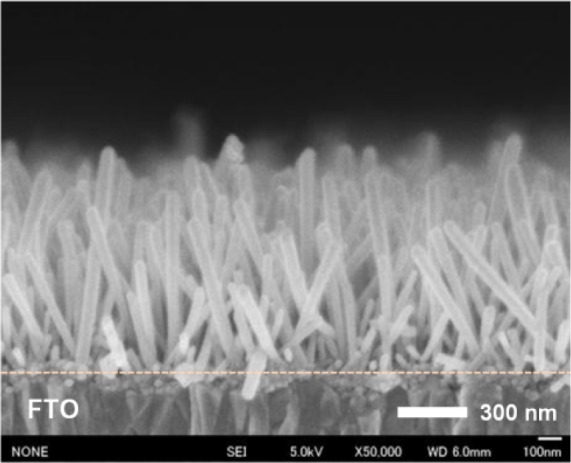
Cross-sectional FESEM image of ZnO NRs grown on 50 nm-thick AZO on the fluorine-doped SnO_2_ (FTO) coated glass substrate. The seed layer annealing temperature is 350 °C.

The average length of the NRs prepared on the FTO coated glass is 760 nm, as shown in Figure 7. It indicates that the growth rate of the NRs at a fixed seed layer annealing temperature is less related to the substrate material [31].

**Figure 8.**
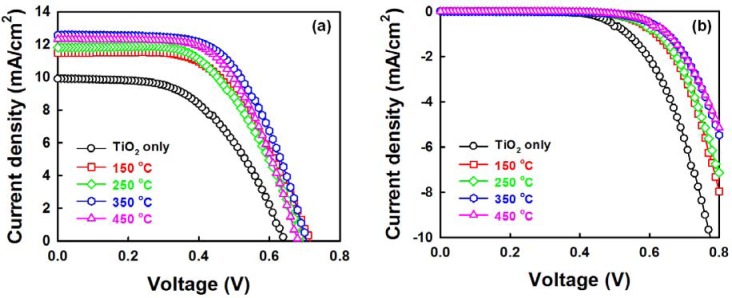
*J-V* curves of dye-sensitized solar cells (DSSCs) with TiO_2_/ZnO NRs under (**a**) illumination and (**b**) dark conditions; the annealing temperature of the TiO_2_ layer was 450 °C for 30 min.

Figure 8a shows the photo current density-voltage (*J-V*) curves of DSSCs fabricated with ZnO NRs grown on the AZO seed layers with various annealing temperatures under air mass 1.5 global (AM 1.5G) illumination. The detailed cell parameters are summarized in Table 1. The cross-sectional FESEM image and schematic diagram of the photoelectrode (TiO_2_/ZnO NRs/FTO) are shown in Figure S2 and XRD pattern of the photoelectrode is shown in Figure S3. The DSSC with ZnO NRs grown on an AZO seed layer annealed at 350 °C shows excellent performance, with a short-circuit current density (*J*_SC_) of 12.56 mA/cm^2^, an open-circuit voltage (*V*_OC_) of 0.70 V, a fill factor (*FF*) of 0.59 and a consequent power conversion efficiency (PCE, η) of 5.20%. The efficiency of the cells with only TiO_2_ is 3.49%. The addition of ZnO NRs therefore results in a 48% enhancement in the cell efficiency. The clear improvement in cell performance is induced by the improved properties of the ZnO NRs. At a seed layer annealing temperature of 350 °C, the improved *J*_SC_ could be considered the result of: (1) the increase in the amount of the dye absorbed on the ZnO NRs; and (2) the fast carrier-transfer process through the NRs to the FTO electrode [32–34]. The amount of dye absorbed on the ZnO NRs increases in proportion to the increase in NR length, leading to the increased light harvesting efficiency. Furthermore, it was reported that the ZnO nanowires (NWs) could increase electron lifetime and decrease electron recombination with the electrolyte in the DSSCs based on the ZnO NWs/TiO_2_ NRs hybrid film [22,32–34].

As shown in Figure 8b, the onset of the dark current shifted to a higher forward bias in the presence of the ZnO NRs. This supports well that the recombination reaction between the FTO and the triiodide ions in the electrolyte is effectively suppressed, which gives rise to a slight increase in the *FF* from 0.53 to 0.59 [35,36]. Meanwhile, the *J*_SC_ and *FF* of the cell are somewhat lower than those reported results on cell-based ZnO nanowires in mesoporous TiO_2_ (*J*_SC_ of 14.72 mA/cm^2^, *FF* of 0.68) [22]. It could be expected that optimizing conditions (electrolyte, counter electrode, *etc*.) will lead to further improvement in the photovoltaic performance of DSSCs.

## 3. Experimental Section

The AZO seed layers were prepared using zinc acetate dihydrate (Zn(CH_3_COOH)_2_·2H_2_O, 0.15 M, Wako, Japan) as the precursor dissolved in ethanol, 2-methoxyethanol (ME) (CH_3_OCH_2_CH_2_OH, Wako, Japan) and Milli-Q solvents. Aluminum nitrate nonahydrate (Al(NO_3_)_3_·9H_2_O, Sigma-Aldrich, Japan) was used as the dopant with an Al/Zn atomic ratio of 1 at%. After stirring at 60 °C for 1 h, the sol was aged at room temperature for 24 h. After spin-coating of the sol on the glass or FTO coated glass substrates, the samples were annealed at different temperatures ranging from 150 to 450 °C for 30 min in ambient air. The thicknesses of the seed layers were 20 nm and 50 nm.

The ZnO NRs were fabricated using various seed layer conditions by a chemical solution deposition with an aqueous solution of zinc nitrate hexahydrate (Zn(NO_3_)_2_·6H_2_O, Sigma-Aldrich, 0.01 M) and hexamethylenetetramine (HMT) (C_6_H_12_N_4_, Sigma-Aldrich, 0.01 M) [30]. The seed layers were immersed in the solution for 3 h at 90 °C to grow the ZnO NRs. The samples were then washed by deionized water and dried at 120 °C for 10 min.

The structural properties of the ZnO seed layer and NRs were examined by X-ray diffraction (XRD, Bruker, D8ADVANCE) with Cu Kα radiation operating at a voltage of 40 keV and a current of 40 mA. The surface morphologies were investigated by atomic force microscopy (AFM, SHIMADZU, SPM-9500J3) and field emission scanning electron microscopy (FESEM, JSM-6701F).

The DSSCs were fabricated as follows [37]. A 4 μm-thick TiO_2_ nanoporous layers on the ZnO NRs was prepared by the screen-printing method and then immersed in an ethanol solution of di-tetrabutylammoniumcis-bis(isothiocyanato)bis(2,2′-bipyridyl-4,4′-dicarboxylato)ruthenium(II) (N719, Sigma-Aldrich, 3 × 10^−4^ M) dye for 3 h at 80 °C. The conducting polymer counter electrode, a 15 nm-thick poly(3,4-ethylenedioxythiophene)-tetramethacrylate (PEDOT-TMA, Sigma-Aldrich), was prepared by a spin-coating method on FTO coated glass substrate. An electrolyte containing lithium iodide (LiI, Sigma-Aldrich, 0.5 M), iodine (I_2_, Sigma-Aldrich, 0.05 M) and 4-*tert*-butylpyridine (TBP, Sigma-Aldrich, 0.05 M) in acetonitrile/polyethylene glycol (4:1 *v/v*) was injected between the two prepared electrodes assembled with a 50 μm-thick spacer. The current density-voltage characteristics of the DSSCs were measured using a Keithley 2400 Source Meter under AM 1.5G (100 mW/cm^2^). The cell area was 0.15 cm^2^.

## 4. Conclusions

The seed layer thickness and Al dopant had significant effects on the growth of ZnO NRs. Up to a certain annealing temperature, the alignment and length of the ZnO NRs were gradually improved with increasing seed layer annealing temperature, due to the enhanced crystallinity and morphology of the seed layer. However, there was no significant difference in the growth of the ZnO NRs on the different substrate materials. When the ZnO NRs grown on an AZO seed layer annealed at 350 °C were inserted between the TiO_2_ and the FTO electrode, the cell exhibited an improved conversion efficiency of 5.20% in comparison with the cells with only TiO_2_ (η, 3.49%).

## Figures and Tables

**Table 1.** Detailed cell parameters of DSSCs employing the TiO_2_/ZnO NRs with various seed layer annealing temperatures under AM 1.5G illumination.

Temperature (°C)	*J*_SC_ (mA/cm^2^)	*V*_OC_ (V)	*FF* (−)	*H* (%)
only TiO_2_	10.28	0.64	0.53	3.49
150	11.48	0.71	0.55	4.48
250	11.80	0.69	0.55	4.47
350	12.56	0.70	0.59	5.20
450	12.32	0.69	0.60	5.08

## Supplementary Materials

Supplementary File 1

## References

[1] Janotti A., van de Walle C.G. (2009). Fundamentals of zinc oxide as a semiconductor. Rep. Prog. Phys.

[2] Kumar B., Kim S.-W. (2012). Energy harvesting based on semiconducting piezoelectric ZnO nanostructures. Nano Energy.

[3] Sun Y., Seo J.H., Takacs C.J., Seifter J., Heeger A.J. (2011). Inverted polymer solar cells integrated with a low-temperature-annealed sol-gel-derived ZnO film as an electron transport layer. Adv. Mater.

[4] Park K., Lee D.-K., Kim B.-S., Jeon H., Lee N.-E., Whang D., Lee H.-J., Kim Y.J., Ahn J.-H. (2010). Stretchable, transparent zinc oxide thin film transistors. Adv. Funct. Mater.

[5] Tsay C.-Y., Fan K.-S., Wang Y.-W., Chang C.-J., Tseng Y.-K., Lin C.-K. (2010). Transparent semiconductor zinc oxide thin film deposited on glass substrates by sol-gel process. Ceram. Int.

[6] Wang X., Song J., Summers C.J., Ryou J.H., Li P., Dupuis R.D., Wang Z.L. (2006). Density-controlled growth of aligned ZnO nanowires sharing a common contact: A simple, low-cost, and mask-free technique for large-scale applications. J. Phys. Chem. B.

[7] Fang T.-H., Kang S.-H. (2009). Surface and physical characteristics of ZnO:Al nanostructured films. J. Appl. Phys.

[8] Caglar Y., Arslan A., Ilican S., Hür E., Aksoy S., Caglar M. (2013). Preparation and characterization of electrodeposited ZnO and ZnO:Co nanorod films for heterojuction diode applications. J. Alloys Comp.

[9] Kung C.Y., Young S.L., Kao M.C., Chen H.Z., Lin J.H., Lin H.H., Horng L., Shih Y.T. Thickness effect of sputtered ZnO seed layer on the electrical properties of Li-doped ZnO nanorods and application on the UV photodetector.

[10] Greene L.E., Law M., Tan D.H., Montano M., Goldberger J., Somorjai G., Yang P. (2005). General route to vertical ZnO nanowire arrays using textured ZnO seeds. Nano Lett.

[11] Elias J., Tena-Zaera R., Lévy-Clément C. (2007). Electrodeposition of ZnO nanowires with controlled dimensions for photovoltaic applications: Role of buffer layer. Thin Solid Films.

[12] Ghayour H., Rezaie H.R., Mirdamadi Sh., Nourbakhsh A.A. (2011). The effect of seed layer thickness on alignment and morphology of ZnO nanorods. Vacuum.

[13] Ji L.-W., Peng S.-M., Wu J.-S., Shih W.-S., Wu C.-Z., Tang I.-T. (2009). Effect of seed layer on the growth of well-aligned ZnO nanowires. J. Phys. Chem. Solids.

[14] Li C., Fang G., Li J., Ai L., Dong B., Zhao X. (2008). Effect of seed layer on structural properties of ZnO nanorod arrays grown by vapor-phase transport. J. Phys. Chem. C.

[15] Wang M., Lee K.E., Hahn S.H., Kim E.J., Kim S., Chung J.S., Shin E.W., Park C. (2007). Optical and photoluminescent properties of sol-gel Al-doped ZnO thin films. Mater. Lett.

[16] Oral A.Y., Bahşi Z.B., Aslan M.H. (2007). Microstructure and optical properties of nanocrystalline ZnO and ZnO:(Li or Al) thin films. Appl. Surf. Sci.

[17] Ozer N., Lampert C.M. (1998). Electrochromic characterization of sol-gel deposited coatings. Sol. Energy Mater. Sol. Cells.

[18] Ilican S., Caglar Y., Caglar M. (2008). Preparation and characterization of ZnO thin films deposited by sol-gel spin coating method. J. Optoelectron. Adv. M.

[19] Guo M., Diao P., Cai S. (2005). Hydrothermal growth of well-aligned ZnO nanorod arrays: Dependence of morphology and alignment ordering upon preparing conditions. J. Solid State Chem.

[20] Gonzalez-Valls I., Lira-Cantu M. (2009). Vertically-aligned nanostructures of ZnO for excitonic solar cells: A review. Energy Environ. Sci.

[21] Anta J.A., Guillén E., Tena-Zaera R. (2012). ZnO-based dye-sensitized solar cells. J. Phys. Chem. C.

[22] Yang G., Miao C., Bu Z., Wang Q., Guo W. (2013). Seed free and low temperature growth of ZnO nanowires in mesoporous TiO_2_ film for dye-sensitized solar cells with enhanced photovoltaic performance. J. Power Sources.

[23] Bai Y., Yu H., Li Z., Amal R., Lu G.Q., Wang L. (2012). *In situ* growth of a ZnO nanowire network within a TiO_2_ nanoparticle film for enhanced dye-sensitized solar cell performance. Adv. Mater.

[24] Giri P.K., Dhara S., Chakraborty R. (2010). Effect of ZnO seed layer on the catalytic growth of vertically aligned ZnO nanorod arrays. Mat. Chem. Phys.

[25] Song J., Lim S. (2007). Effect of seed layer on the growth of ZnO nanorods. J. Phys. Chem. C.

[26] Kakati N., Jee S.H., Kim S.H., Oh J.Y., Yoon Y.S. (2010). Thickness dependency of sol-gel derived ZnO thin films on gas sensing behaviors. Thin Solid Films.

[27] Seto J.Y.W. (1975). The electrical properties of polycrystalline silicon films. J. Appl. Phys.

[28] Qu X., Jia D. (2009). Controlled growth and optical properties of Al^3+^ doped ZnO nanodisks and nanorod clusters. Mater. Lett.

[29] Wahid K.A., Lee W.Y., Lee H.W., Teh A.S., Bien D.C.S., Azid I.A. (2013). Effect of seed annealing temperature and growth duration on hydrothermal ZnO nanorod structures and their electrical characteristics. Appl. Surf. Sci.

[30] Polsongkram D., Chamninok P., Pukird S., Chow L., Lupan O., Chai G., Khallaf H., Park S., Schulte A. (2008). Effect of synthesis conditions on the growth of ZnO nanorods via hydrothermal method. Phys. B.

[31] Kim K.H., Umakoshi T., Abe Y., Kawamura M. (2014). Growth of zinc oxide nanorods using various seed layer annealing temperature and substrate materials. Int. J. Electrochem. Sci.

[32] Umar A.A., Rahman M.Y.A., Taslim R., Salleh M.M., Oyama M. (2012). Effect of the thickness of quasi one-dimensional zine oxide nanorods synthesized via multiple growth process under ammonia assisted hydrolysis technique on the performance of dye-sensitized solar cell. Int. J. Electrochem. Sci.

[33] Pang S., Xie T., Zhang Y., Wei X., Yang M., Wang D., Du Z. (2007). Research on the effect of different sizes of ZnO nanorods on the efficiency of TiO_2_-based dye-sensitized solar cells. J. Phys. Chem. C.

[34] Yun S., Lee J., Chung J., Lim S. (2010). Improvement of ZnO nanorod-based dye-sensitized solar cell efficiency by Al-doping. J. Phys. Chem. Solids.

[35] Wu S., Han H., Tai Q., Zhang J., Xu S., Zhou C., Yang Y., Hu H., Chen B., Sebo B., Zhao X.-Z. (2008). Enhancement in dye-sensitized solar cells based on MaO-coated TiO_2_ electrodes by reactive DC magnetron sputtering. Nanotechnology.

[36] Koide N., Islam A., Chiba Y., Han L. (2006). Improvement of efficiency of dye-sensitized solar cells based on analysis of equivalent circuit. J. Photochem. Photobiol. A.

[37] Kim K.H., Utashiro K., Jin Z., Abe Y., Kawamura M. (2013). Dye-sensitized solar cells with sol-gel processed Ga-doped ZnO passivation layer. Int. J. Electrochem. Sci.

