# Supplementary material for: Structural Properties of Zinc Oxide Nanorods Grown on Al-Doped Zinc Oxide Seed Layer and Their Applications in Dye-Sensitized Solar Cells
Source: Materials (Basel). 2014 Mar 28;7(4):2522–33. doi: 10.3390/ma7042522 (PMC5453348; doi:10.3390/ma7042522)
Supplement: Supplementary File 1 [file materials-07-02522-s001.pdf]

Supplementary Information

Table S1. Zn2p[3], O1s, C1s, and Zn/O ratio calculated from XPS spectra.

**Film**	**Zn2p**		**O1s**		**C1s**		**Zn/O**
	**Peak (eV)**	**at%**	**Peak (eV)**	**at%**	**Peak (eV)**	**at%**	
ZnO	1022.44	37.98	531.17	41.26	285.90	20.75	0.92
AZO	1022.42	44.14	531.17	45.40	285.84	10.45	0.97

Note: Film thickness: 50 nm, Annealing temperature: 350 °C.

Figure S1. (**a**) XRD patterns and (**b**) relative intensity of (002) peak of the ZnO NRs grown on AZO seed layers with various seed annealing temperatures.

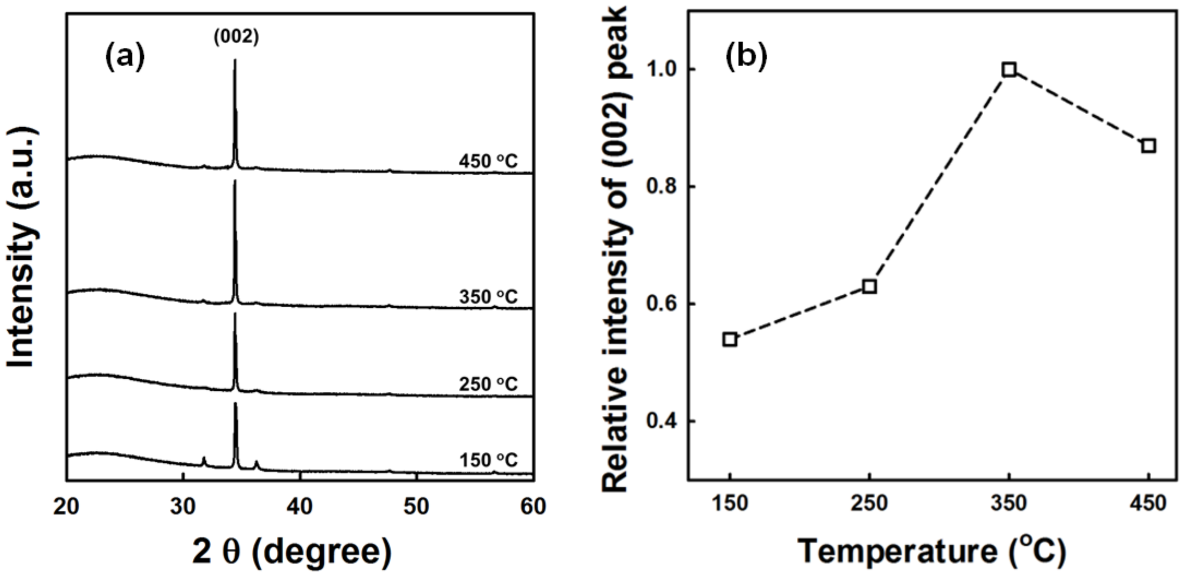

Figure S2. Cross-sectional FESEM images of (**a**) TiO_2/ZnO NRs on FTO glass coated substrate and (**b**) its schematic diagram.

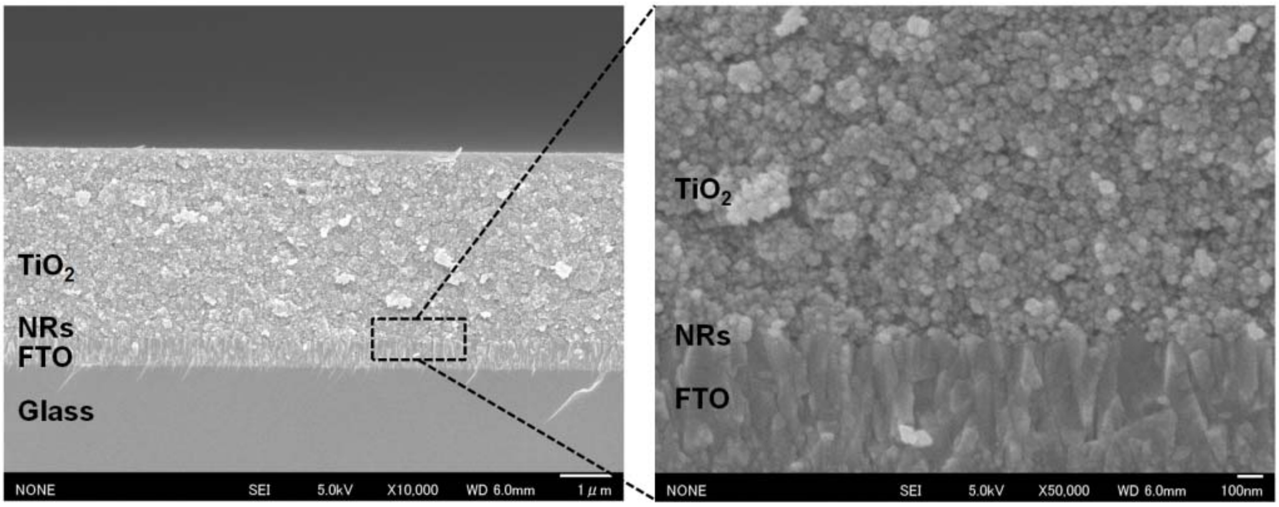

(**a**)

Figure S2. *Cont.*

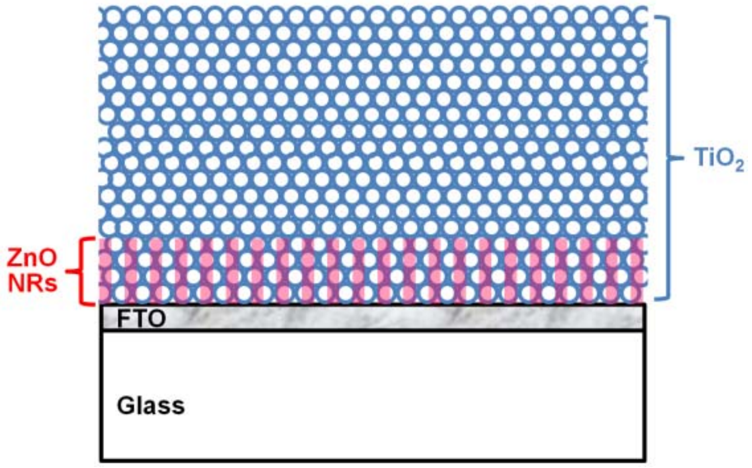

(**b**)

As seen, TiO_2 nanoparticles could effectively fill in the interspaces between ZnO nanorods.

Figure S3. XRD pattern of TiO_2/ZnO NRs on FTO coated glass substrate.

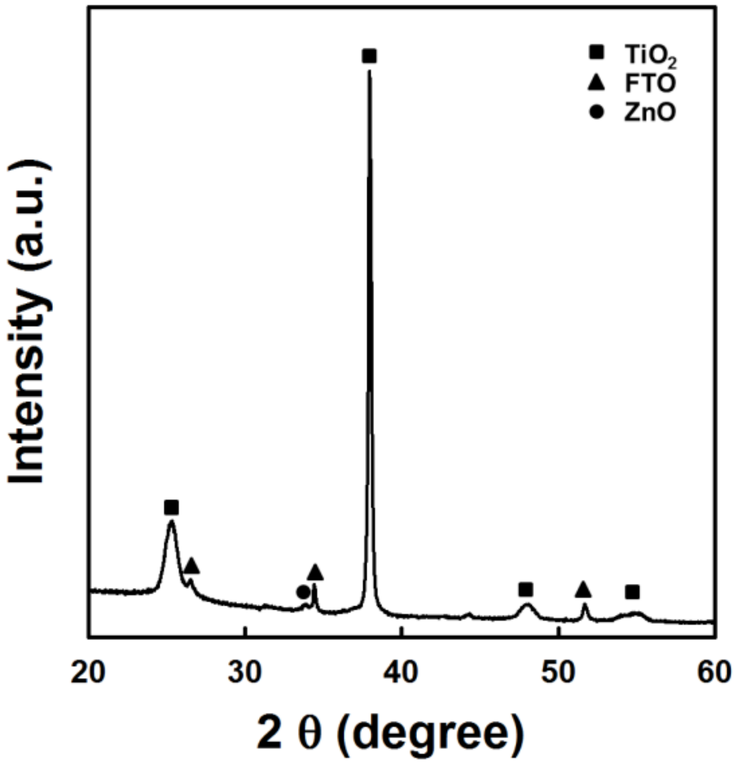

Diffraction peak corresponding to the hexagonal wurtzite structure of ZnO is clearly observed.
